# Orthopaedic surgeons' perceptions and attitudes on COVID-19 related changes in practice: an international cross-sectional survey

**DOI:** 10.11604/pamj.2021.38.96.26846

**Published:** 2021-01-28

**Authors:** Nadhir Meraghni, Hichem Bouyoucef, Redouane Si Larbi, Nacim Soal, Riad Benkaidali, Mohamed Derradji, Zoubir Kara, Amine Hamza

**Affiliations:** 1 Department of Orthopaedic Surgery, Centre Hospitalier Universitaire Mustapha Bacha, Algiers, Algeria,; 2 Department of Orthopaedic Surgery, Centre Hospitalier Sainte Catherine, Saverne, France,; 3 The Ouarsenis Medical Center, Algiers, Algeria,; 4 Department of Orthopaedic Surgery, Centre Hospitalier du Mans, Le Mans, France

**Keywords:** COVID-19, orthopaedic surgery, elective surgery

## Abstract

**Introduction:**

the purpose of this study was to assess the orthopaedic surgeons' perceptions and attitudes on COVID-19 related changes in their practice.

**Methods:**

an online survey was shared with orthopaedic surgeons practicing in different countries.

**Results:**

this study showed that orthopaedic surgery plan management was adapted to respond more effectively to the COVID-19 pandemic while maintaining the continuity of health care and ensuring protection of medical staff and patients. Among the introduced measures, elective surgery was postponed to free-up beds for suspected or COVID-19 positive patients requiring hospitalization. Additionally, the number of outpatient visits was considerably decreased and non-urgent visits were postponed to reduce the flow of patients in and out of hospitals and therefore minimize the risk of contamination. Interestingly, data revealed the willingness of orthopaedic surgeons to take care of COVID-19 positive patients and support their colleagues in intensive care units, if needed.

**Conclusion:**

orthopaedic surgery departments have adapted their programs to face the unprecedented challenges due to the COVID-19 pandemic. Quick measures were taken to reduce the risk of contamination in patients, medical staff and to allow hospitals to free up beds for treatment of patients with positive or suspected COVID-19.

## Introduction

The COVID-19 pandemic is one of the largest global healthcare crises in nearly a century. The novel coronavirus crisis started in Wuhan, China in late 2019 and has spread worldwide [1]. On the 11^th^ of March 2020, the World Health Organization (WHO) declared that the epidemic of COVID-19 had become a pandemic [2]. To face this global health emergency which has overwhelmed the health systems around the world, health institutions have had to readjust their functioning, to cope with COVID-19 while ensuring the continuity of care and protecting medical staff and patients [3]. At first glance, orthopaedic surgeons are not considered front-line staff in the fight against the COVID-19, comparing with our colleagues of other specialities: infectiologists, pneumologists and intensive care physicians. However, as part of the larger healthcare system, they also have an important role to play in reining in this pandemic [1-4]. The purpose of this study was to assess the orthopaedic surgeons´ perceptions and attitudes on COVID-19 related changes in their practice.

## Methods

We developed an online, anonymous web-based survey using non-probability snowball sampling technique. A 29-question anonymous survey (Annex 1) was shared with orthopaedic surgeons practicing in different countries across social media orthopaedic groups and platforms. The questionnaire was designed to assess the orthopaedic surgeons´ perceptions on COVID-19 related changes in their practice. We shared the online questionnaire and collected relevant data. Results were exported to Microsoft Excel version 2010 for analysis. The survey included different sections. The first one was about general information: country of practice, age, sector of activity and years of experience. Then, we asked about knowledge about the COVID-19: specific training, recommendations, risks and protection. The respondents were required to report their experience of care for COVID-19 positive patients and their motivation to work in COVID-19 units. Specific questions were asked about orthopaedic and trauma activity: number of surgeries (planned or emergency), number of outpatient visits and supply of orthopaedic equipment. They were also interrogated if they had reported symptoms or had suspected a COVID-19 infection and what were the measures taken about that. They were also required to report the level of personal protection at work, which equipment they used and which protection equipment is missing the most in their institution. Finally, the respondents were asked about their countries strategy and what do they think is the best solution to face the COVID-19 pandemic.

## Results

**Figure 1:**
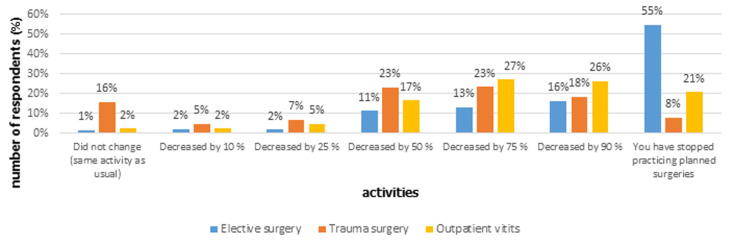
impact on the elective surgery, trauma surgery and outpatient visits

Five hundred and thirty four (534) orthopaedic surgeons representing 80 countries (Table 1, Table 1 (suite)) participated to the study. The mean age of participants was 40 ± 9 years (minimum 29 years, maximum 76 years old). Fifty nine percent (59%) are working in public hospitals. Twenty one percent (21%) and 19% of participants have more than 10 and 20 years´ experience in orthopaedic surgery, respectively. Sixty percent (60%) of orthopaedic surgeons reported having extensive knowledge of COVID-19 and 53% have confirmed having received relevant training at their place of work. Four hundred and twenty five (425) surgeons asked (79%) reported their awareness about measures of protection and risks for medical staff and patients. Seventy five (75) respondents (14%) reported having operated on COVID-19 positive patients but 52% declared their willingness to operate suspected or COVID-19 positive patients with orthopaedic pathology. Sixty two percent (62%) of orthopaedic surgeons declared being ready to work and help in intensive care units if needed. Concerning their specific activity, 55% have cancelled elective surgeries while 16% reduced their activity by about 90%. The number of emergency and trauma surgeries has decreased for the majority of physicians asked, by 90% for 18% and by 50% for 23% of them. Sixteen percent (16%) of surgeons declared having the same trauma activity as usual (Figure 1). In the same way, more than 59% notified a clear decrease (more than 75%) on the number of traffic accidents. Twenty six percent (26%) reduced the number of outpatient visits by 90% while 113 surgeons (21%) have cancelled all outpatient visits. The supply of orthopaedic equipment for the orthopaedic departments was not affected according to 60% of respondents. Three hundred and thirty eighty (338) doctors (63%) have used telemedicine with their patients during the COVID-19 pandemic. One hundred and forty seven (147) surgeons (27%) experienced one or many symptoms suspecting a COVID-19 infection. The best option was to stay home and observe their symptoms for 51% of them. Sixty two percent (62%) of respondents reported self-isolating at home when they return back from work place. Seventy four percent (74%) declared feeling stressed and anxious about this global pandemic. The equipment used for personal protection are medical masks, respirator N 95 or FFP2 masks, face shields, gloves, gowns and protective glasses. Seventy seven percent (77%) of respondents estimated that their countries were under-equipped to deal with the COVID-19 pandemic while 57% were in agreement with their institutions strategies to face this crisis. To avoid COVID-19, orthopaedic surgeons asked recommend to people: regular hands washing, hydro-alcoholic sanitizer, social distancing, face masks, gloves and of course to stay home. Finally, 37% of the interviewed doctors believe that the best solution for COVID-19 would be to make a specific vaccine while 37% proposed respect of quarantine as a preventive method to face the COVID-19 pandemic.

**Table 1:** list of countries

Country	Responses
Albania	1
Algeria	185
Andorra	1
Argentina	2
Australia	2
Austria	2
Bangladesh	3
Barbados	1
Belgium	27
Brazil	18
Burkina Faso	3
Cameroon	1
Canada	3
Central African Republic	1
Colombia	3
Democratic Republic of the Congo	1
Cote d'Ivoire	3
Croatia	4
Egypt	6
El Salvador	1
Finland	6
Georgia	1
Germany	3
Hong Kong	1
Hungary	1
India	57
Indonesia	4
Iran	4
Iraq	1
Ireland	1
Italy	7
Japan	4
Kenya	3
Latvia	1
Lebanon	1
Libya	2
Luxembourg	4
Macedonia	1
Madagascar	2
Malaysia	1
Mali	1
Mauritania	1
Mexico	1
Moldova	2
Monaco	1
Morocco	9
Namibia	1
Nepal	9
New Zealand	1
Nigeria	5
Oman	3
Pakistan	6
Paracel Islands	1
Paraguay	10
Philippines	4
Poland	1
Portugal	2
Qatar	1
Romania	14
Russia	5
Saudi Arabia	3
Senegal	4
Singapore	2
South Africa	2
Spain	1
Sudan	1
Sweden	6
Switzerland	11
Syria	1
Taiwan	2
Togo	2
Tunisia	12
Turkey	2
Uganda	2
Ukraine	1
United Kingdom	17
United States	11
Uzbekistan	1
Vietnam	1
Yemen	1
Total	**534**

## Discussion

All elective surgical procedures should be cancelled and deferred until an opportune time [5,6]. Trauma cases surgeries should continue to proceed. Intraoperatively, full personal protection including surgical shields and goggles should be used. Operative times should be reduced whenever feasible, and surgical team should be kept to the minimum, whenever possible [4,5,7]. The reported decrease in the number of road accidents is due to the lockdown policy imposed by several countries worldwide and remote work adopted by a large number of institutions and organizations. Further measures may also be implemented. Hospitals should be in lockdown with no visitors allowed, social distancing at work (between coworkers) and at home (between cohabiting health-care workers). Physicians have also been advised to prolong the duration between non-urgent follow-ups to reduce patient overcrowding in hospitals [4,5,7]. Although non-urgent clinics and surgical procedures have been postponed until the situation improves, we must ensure that appropriate quality of care given to our patients is maintained. The emergence of such a crisis provides a timely opportunity for us to reflect and evaluate the use of novel technologies in the workplace. This includes the adoption of telemedicine and telerehabilitation initiatives, allowing patients to be consulted and followed-up in the comfort of their own homes [4,5,8-10]. Apart from the information shared by healthcare institutions, healthcare professionals used various other sources of information such as television, social media and World Health Organization website [11].

This study demonstrated high level of awareness amongst orthopaedic surgeons about the risk of infection in healthcare professionals and patients as well as the preventive measures for stopping or minimizing spread of the disease. Given the increased risk for transmission COVID-19 virus in hospitals in general and operating theaters in particular, special personal protective measures must be provided. As surgeons, utmost care must be given to patients in the preoperative, intraoperative, and postoperative settings to minimize the risks of contamination. The risks and benefits of surgical management should be rationalized for each patient [4,11,12]. COVID-19 has shown more infectivity and a higher fatality rate than the H1N1 epidemic [2,11]. In addition, important clinical features of COVID-19 are currently unknown. These two elements may explain the number of interviewees (48%) who expressed reluctance to treat or operate non-urgent conditions in COVID-19 positive patients. Notably, the most common reason for their unwillingness to treat COVID-19 positive patients is due to their concern of getting the infection and transmitting the virus to their family members. With a better understanding of COVID-19 characteristics, we would expect a gradual increase in the number of medical staff who will be willing to treat infected patients [2,11]. The COVID-19 crisis has resulted in people working outside their specialty, providing support to infectiologist, pneumologist and intensive care physicians [1,5]. We know that virus is likely to cause minor symptoms in majority (more than 80%) of infected people. Many healthcare workers are likely to fall into this category [1]. Orthopaedic surgeons have a reputation built on their versatility and strength. Emotional support is necessary for ourselves, colleagues, patients and families [1,13,14]. Most interviewed practitioners estimated appropriate protective measures have been provided at work (Table 2). Provision of personal protective equipment to healthcare professionals has been a huge challenge in many countries [1,7,15].

**Table 2:** degree of personal protection

Degree	Number of respondents	Percentage
1	80	14.87%
2	118	21.93%
3	209	38.85%
4	101	18.77%
5	30	5.58%
Total	538	100

How do you estimate your personal protection against COVID-19 during your medical practice? [Please choose from 1= not protected to 5 = well protected]

Personal protective equipment included medical masks, respirator N 95 or FFP2 masks, face shields, gloves, gowns, and protective glasses [11,16,17]. Given the extent of the pandemic, a shortage of a widely used equipment has been reported with FFP2 masks arrive in the top of the list -reported by 58% of the interviewed participants- (Table 3). Institutions and governments have put in place different strategies to face this shortage, notably, support increasing production capacity and accelerating approval of protective equipment during this crisis [17,18]. To help prevent spread of COVID-19, orthopaedic surgeons recommendations were frequent hand washing for a minimum 20 seconds, use hydro-alcoholic sanitizer, social distancing, use of face masks, gloves and of course confinement while waiting to discover a specific treatment and to make a specific vaccine. This study included surgeons representing 80 countries with different programs and organization, which can explain the differences in findings. Additionally, the severity of the outbreak was not the same in all countries, so the measures taken by governments and health institutions were different [12]. We recognize certain limitations in our study. The data collected are subjective and represent perceptions of orthopaedic surgeons asked. Few countries have enough participants to make meaningful inferences about these responses relevant to each country. Another limitation is that the non-response rate could not be calculated and distribution of responses was skewed. Finally, the experience of the person who fills out the questionnaire may affect the results.

**Table 3:** personal protection equipment (PPE)

PPE	Number of respondents	Percentage
Face shields	66	12.27%
Gloves	7	1.30%
Gowns	33	6.13%
Medical masks	79	14.68%
Protective glasses	42	7.81%
Respirator N 95 or FFP2 masks	311	57.81%
**Total**	538	100

Which personal protection equipment stock your institution is missing the most

## Conclusion

The COVID-19 pandemic put immense pressure on healthcare systems across the world, including orthopaedic practice. Although, the daily activity of orthopaedic surgery has been markedly affected, surgical teams have responded effectively to the COVID-19 pandemic while maintaining the continuity of health care and ensuring protection of medical staff and patients. The severity of the outbreak was not the same in all countries represented in our study which can explain the differences in findings.

### What is known about this topic

- Cancellation of elective surgical procedures after the spread of the COVID-19;
- Negative impact on surgical practice worldwide;
- Need of a safety resumption of elective surgery.

### What this study adds

- Orthopaedic surgery departments have quickly adapted their functioning in response to the emergence of the COVID-19 pandemic;
- Although non-urgent clinics and surgical procedures have been postponed until the situation improves, we must ensure that appropriate quality of care given to our patients must be ensured;
- The emergence of such a crisis provides a timely opportunity for us to reflect and evaluate the use of novel technologies like telemedicine in the workplace.

## Annex

**Annex 1**: survey questions (PDF - 356Kb)
